# Antimicrobial activity of complete denture cleanser solutions based on sodium hypochlorite and *Ricinus communis* – a randomized clinical study

**DOI:** 10.1590/1678-775720150204

**Published:** 2015

**Authors:** Marcela Moreira SALLES, Maurício Malheiros BADARÓ, Carolina Noronha Ferraz de ARRUDA, Vanessa Maria Fagundes LEITE, Cláudia Helena Lovato da SILVA, Evandro WATANABE, Viviane de Cássia OLIVEIRA, Helena de Freitas Oliveira PARANHOS

**Affiliations:** 1 - Universidade de São Paulo, Faculdade de Odontologia de Ribeirão Preto, Departamento de Materiais Dentários e Prótese, Ribeirão Preto, SP, Brasil.; 2 - Universidade de São Paulo, Faculdade de Odontologia de Ribeirão Preto, Departamento de Odontologia Restauradora, Ribeirão Preto, SP, Brasil.

**Keywords:** Biofilms, Denture cleansers, Sodium hypochlorite, Castor oil, Complete denture

## Abstract

To preserve oral health and to maintain the prosthetic devices, it is important not only to improve the properties of commonly known hygiene products, but also to investigate new materials with antimicrobial action.

**Objectives:**

This study evaluated the antimicrobial activity of sodium hypochlorite (0.25% and 0.50%) and 10% *Ricinus communis*’ solutions against specific microorganisms.

**Material and Methods:**

Sixty four maxillary complete denture wearers were instructed to brush their dentures three times a day and to soak them (20 min/day) in the solutions: SH1: 0.25% sodium hypochlorite; SH2: 0.5% sodium hypochlorite; RC: 10% *R. communis* oil; and C: 0.85% saline (control). The solutions were used for 7 days in a randomized sequence. Following each period of use, there was a 1-week washout period. Antimicrobial activity was determined by Colony Forming Units (CFU) counts of *Streptococcus mutans*, *Candida* spp., and gram-negative microorganisms. For collecting biofilm, the internal surface of maxillary dentures was brushed with saline solution, and biofilm suspension obtained. After dilutions (10^0^ - 10^-3^), aliquots were seeded in Mitis salivarius, CHROMagar Candida^®^, and MacConkey agar for detecting *S. mutans*, *Candida* spp., or gram-negative microorganisms, respectively. After incubation, colonies were counted, and CFU/mL values were calculated. Then, transformation - log10 (CFU+1) - data were analyzed using the Friedman test (α=0.05). Results showed significant differences between the solutions (p<0.001).

**Results:**

All three solutions showed antimicrobial activity against *S. mutans*. Against *Candida* spp., RC and SH1 solutions showed similar effect while SH2 showed superior activity. SH1 and SH2 solutions showed antimicrobial action against gram-negative microorganisms. The *Candida* species most frequently isolated was *C. albicans*, followed by *C. tropicalis* and *C. glabrata*.

**Conclusions:**

The 0.5% sodium hypochlorite solution was the most effective and might be used to control denture biofilm. *C. albicans* was the most frequently isolated *Candida* sp.

## INTRODUCTION

Denture biofilms contain microorganisms that can cause local and systemic infections^6^. Although several methods have been indicated to control denture biofilms, studies have shown that chemical cleansers and brushing are the most effective^1^ ^,^ ^22^. Ease of use, ability to remove microorganisms present in the microporosity on the surface of acrylic resins, and incorporation of agents with antimicrobial activity are the main advantages of using chemical cleansers for maintaining oral hygiene^8^. The main disadvantage is the possible adverse effect on the prosthetic device, depending on the immersion time and concentration used^17^ ^,^ ^18^.

Sodium hypochlorite is a chemical solution that is routinely recommended for cleaning dentures. Although it is an effective disinfecting agent^16^ ^,^ ^30^, its use is limited because it whitens acrylic resins^18^ and corrodes metal components of prostheses^7^ ^,^ ^17^. A factor to be pointed out is the importance to evaluate its effectiveness through randomized controlled clinical trials, since most of *in vivo* studies have been developed without standardization^28^.

Oil derived from castor bean *(Ricinus communis)* is used in the medical field because of its biocompatibility and bactericidal and fungicidal effects^11^. In Dentistry, new specific formulations have been developed such as Endoquil, an irrigating solution for root canals^15^, and Perioquil, a mouthwash used in periodontology and prosthodontics^19^. Concerning complete denture cleanser, a 2% *R. communis *oil solution has shown moderate efficacy in removing denture biofilms. Moreover, authors have highlighted the importance of studying other concentrations of *R. communis* oil^1^.

Because of the complex composition and maturation degree of the denture biofilms, it is important to evaluate the antimicrobial activity of cleanser solutions. This property has been evaluated using different methods, but comparing their results is difficult, and there is no consensus on the most effective chemical cleanser. In addition, only few randomized controlled trials have been performed on immersion denture cleansers, and even fewer trials have been performed using adequate methods^28^. Therefore, this crossover randomized clinical trial aimed to evaluate the antimicrobial activity of sodium hypochlorite (0.25% and 0.50%) and *R. communis *oil (10%) solutions along with the mechanical method of brushing against *Streptococcus mutans*, *Candida *spp*., *and gram-negatives. As a complementary analysis, the study identified *Candida* spp. frequently present in denture biofilms and their resistance to main antifungal agents. The null hypothesis tested was that immersion in denture cleansers and a control medium would have the same antimicrobial action against the evaluated biofilm.

## MATERIAL AND METHODS

### Subject selection and solutions

The study was approved by the Institutional Review Board of the School of Dentistry of Ribeirão Preto (FORP/USP) (Brazilian Ethics System register number, CAAE 0007.0.138.000-08) and obtained a signed informed consent form from the participants. Seventy-six individuals, who visited the Complete Denture Clinic of FORP/USP for new complete dentures confection, during a school semester, were evaluated for possible participation in this study. Inclusion criteria comprised: adult patients, both genders, any age, good general health, complete edentulism and wearing of, at least, maxillary complete dentures fabricated by heat-polymerized acrylic resin and acrylic artificial teeth. The exclusion criteria were: dentures that had been used for less than 01 year, as well as the relined, repaired, or fractured ones.

Participants were instructed to brush their dentures three times a day (after breakfast, lunch, and dinner) with a specific brush (Bitufo^®^, Itupeva, SP, Brazil) and neutral liquid soap (Pleasant, Perol Commercial and Industrial Ltda., Ribeirão Preto, SP, Brazil), and to soak the dentures (for 20 min) once a day in 200 mL of the following solutions: SH1: 0.25% sodium hypochlorite (Inject Center, Ribeirão Preto, SP, Brazil); SH2: 0.5% sodium hypochlorite (Inject Center); RC: 10% *R. communis* oil solution (Institute of Chemistry, University of São Paulo, São Carlos, SP, Brazil); and C: 0.85% saline solution (control; sodium chloride P.A.; Labsynth Laboratory Products Ltda., Diadema, SP, Brazil). All participants used each solution for 7 days in a random sequence. Following each period of use, there was a 1-week washout period during which the patients used the specific brush and neutral liquid soap to clean their dentures.

### Microbiological analysis

Biofilms were collected from the maxillary dentures of each participant at five time points, i.e., before using the solutions (baseline) and after using them. Before using each solution, the internal surfaces were stained by a disclosing solution (1% neutral red), cleaned by researchers with a specific brush (Bitufo^®^, Itupeva, SP, Brazil) and neutral liquid soap (Pleasant, Perol Commercial and Industrial Ltda., Ribeirão Preto, SP, Brazil), and then returned to the patients in the same clinical initial condition. Biofilms were collected in an aseptic zone by placing each complete maxillary denture in a sterile Petri dish. Dentures were rinsed with 10 mL saline solution, and their internal surfaces were brushed (Tek, Johnson & Johnson Brazil’s Industry and Commerce Healthcare Products Ltda., São José dos Campos, SP, Brazil) for 2 min. The biofilm suspension obtained was vortexed for 2 min and diluted in decimal series (10^0^ - 10^-3^). Then, 50 µL aliquots from the decimal dilutions were cultured in Petri dishes containing Mitis salivarius agar base (more bacitracin solution and 20% sucrose), MacConkey agar, and CHROMagar^®^ Candida for detecting *S. mutans*, gram-negative microorganisms, and* Candida* spp., respectively. Culture media were incubated at 37°C according to each respective condition: candle jar for 48 - 72 h (*mutans* group), or aerobiosis for 48 h (gram-negative and *Candida *spp).

After incubation, the number of colonies for each dilution was counted. Based on the dilution that provided 1 - 300 colonies, colony forming units (CFUs) were determined using the formula CFU/mL=number of colonies x 10^n^/q, where “n” is the absolute value of the dilution (0, 1, 2, or 3) and “q” is the quantity of plated suspension (0.05 mL).

For *Candida* spp., the number of colonies was counted and the species were identified based on the chromogenic properties of the medium. Each identified species was confirmed using yeasts kit (Candifast^®^, ELITech Microbio, Signes, France); in addition, the resistance to antifungal agents, such as amphotericin B, nystatin, flucytosine, econazole, ketoconazole, miconazole, and fluconazole, was evaluated.

In order to conceal the ones involved, the solutions were dispensed in identical dark flasks and delivered without specific identification, and in the quantity to be used for a period of seven days. Each cleanser solution was used by the participants in a random sequence. Researcher P1, who was not involved in the other operational phases of the study, used a computer program to obtain a list of random numbers corresponding to the possible sequences of the treatment. Researcher P2 received these numbers and distributed the solutions to the participants. Researcher P3 implemented hygiene instructions and collected the prostheses. Researcher P4 collected the biofilms while researcher P5 washed the prostheses to ensure the complete elimination of the biofilms. Researcher P2 obtained variable information and provided it to researcher P1, who performed statistical analyses. Thus, during the study, all researchers, as well as the participants, were blinded to the applied solutions.

### Data analysis

The data (CFU+1) were transformed in log10. The values did not adhere to normal distribution, as verified using Kolmogorov Smirnov test. The test solutions were compared using Friedman test (α=0.05), followed by multiple comparisons with Wilcoxon test (α=0.005), and were corrected using Bonferroni method. All statistical tests were performed using SPSS 17.0 software (SPSS Inc., Chicago, IL, USA).

## RESULTS

Seventy-six participants were invited to participate in the study (screening). Of these, five participants were excluded because they did not wear maxillary complete dentures. Of the remaining 71 participants, four did not properly attend to the scheduled returns due to health problems, one discontinued the treatment, and two withdrew from the study because of improper use of the solutions and hence were excluded from statistical analyses. Thus, the final study sample comprised 64 participants (14 men and 50 women) with a mean age of 68 years.

**Figure 1:**
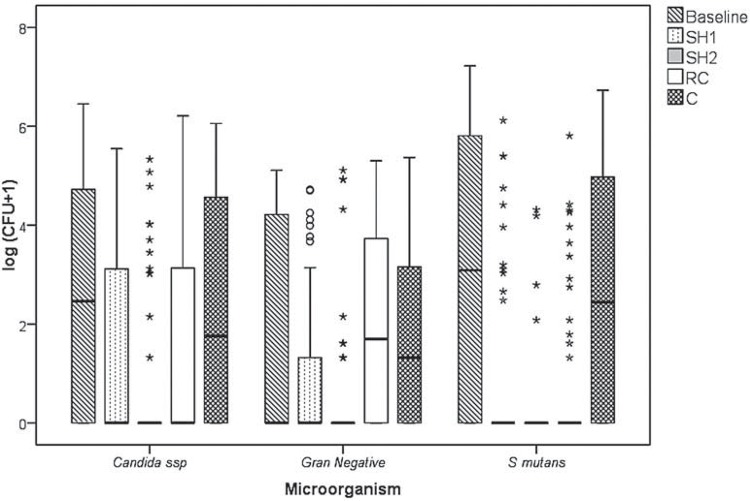
Log (CFU/mL+1) of *Candida* spp., gram-negative microorganisms and *S. mutans* counts at baseline and after using the solutions

Figure 1 shows the CFU/mL values transformed into log10 (CFU+1).

Friedman test showed a significant difference between the test solutions (p<0.001). Table 1 summarizes the mean posts and the results of multiple comparison.

**Table 1:** Mean posts (MP) and results of Friedman test

	Gram-negative		*S. mutans*		*Candida* spp.	
Solutions	MP	Fr (P - Value)	MP	Fr (P - Value)	MP	Fr (P - Value)
Baseline	3.37^A^	53.57 (<0.001)*	3.84^A^	75.16 (<0.001)*	3.54^A^	42.73 (<0.001)*
SH1	2.45^B^		2.63^B^		2.65^CD^	
SH2	2.30^B^		2.37^B^		2.37^D^	
RC	3.46^A^		2.63^B^		3.03^BC^	
C	3.41^A^		3.54^A^		3.41^AB^	

* Significant difference (P<0.05). Comparison between pairs: different letters indicate a significant difference

Regarding the *Candida* spp., *R. communis* solution presented similar action to 0.25% sodium hypochlorite (p=0.155), with results inferior to 0.5% hypochlorite (p=0.003). For gram-negative microorganisms, the 0.25% and 0.5% sodium hypochlorite solutions showed similar action (p<0.001), while the *R. communis* solution proved to be ineffective (p=0.574). The results showed antimicrobial activity of three tested solutions on *S. mutans* (p<0.001).

During the study, *C. albicans*,* C. glabrata*,* C. tropicalis*, and* C. parapsilosis* were the most frequently isolated *Candida* spp. from denture biofilms. *C. albicans* was the most frequently detected *Candida* sp. in biofilms isolated at baseline and after the use of cleanser solutions, followed by *C. tropicalis* and *C. glabrata*.

Of the 159 isolated strains, only 18 (11.3%) were resistant to at least one of the tested antifungals, highlighting their resistance to azole compounds. None of the isolated strains was resistant to polyene compounds.

## DISCUSSION

This study evaluated the antimicrobial action of alkali hypochlorite and *R. communis* solutions through a randomized clinical trial. Both methods (brushing and soaking) were employed in a standardized way. Test solutions were used in a crossover configuration and in a randomized sequence. We tried to minimize bias and blind the researchers, participants, and statisticians whenever possible. The dentures were brushed with a neutral soap, an auxiliary agent with no antimicrobial or therapeutic properties. Both solutions were employed at room temperature (23±2°C) for 20 min, since denture wearers are reluctant to stay without the dentures for prolonged periods and an ideal cleanser must show efficacy in short periods of immersion^8^.

Antimicrobial activities of both sodium hypochlorite and *R. communis* oil solutions were evaluated against* Candida *spp., which cause denture stomatitis^24^; *S. mutans*, which colonize prosthetic surfaces^29^ and cause denture stomatitis; and gram-negative microorganisms, which are not normal microbiota of the oral cavity and cause bacteremia because of biofilm formation^27^.

Results showed that the test solutions affected the microbial count, as evidenced by a reduction in CFU after the first visit (baseline). In addition, effective brushing, particularly with specific brushes, and participants’ motivation to maintain oral hygiene may have contributed to this reduction^28^.

Clinical studies have reported the antimicrobial activity and denture biofilm removal capacity of 1% - 5.25% sodium hypochlorite solutions^1^ ^,^ ^10^. This efficacy has been related to the solvent action as well as to the bactericidal and fungicidal properties of this solution. In this study, we used 0.25% and 0.5% sodium hypochlorite solutions to avoid adverse effects on acrylic resin. However, immersion in a 0.5% solution, employed for 20 min a day, did not cause clinical significant alterations on the color, surface roughness, and flexural strength of an acrylic resin in a simulated period of 5 years of use^2^.

Our findings showed that 0.25% and 0.5% sodium hypochlorite solutions were effective and significantly reduced CFUs compared with saline (control). These results are relevant, since it shows, by a randomized clinical trial, the efficacy of these concentrations when employed in short immersions (20 min). The 0.5% sodium hypochlorite solution showed excellent effectiveness and eliminated the evaluated microorganisms, since it was observed that there was no growth of CFU after its use by almost all the patients. Studies employing protein evaluation^13^ and biofilm disclosure procedures^23^ for biofilm quantification have shown the adequate capacity of 0.5% sodium hypochlorite solution to remove denture biofilms; however, none of these studies have evaluated the antimicrobial activity of this solution. Porta, et al.^22^ (2013), through microbiological clinical assay, found that soaking dentures for 3 min in 0.5% NaOCl for 90 days was an efficacious treatment against *Candida *spp. and pointed out the antimicrobial activity of NaOCl on essential enzymatic sites in bacteria, promoting their irreversible inactivation via the action of hydroxyl ions and chloramination. However, other species were not evaluated.

Similarly, 0.25% sodium hypochlorite solution also eliminated *S. mutans* and was effective against gram-negative microorganisms. However, 0.25% sodium hypochlorite solution showed moderate activity against *C. albicans*, which was similar to th*R. communis *oil solution. Clinical studies involving denture wearers showed antimicrobial activity of 0.02% and 0.05% sodium hypochlorite solutions against *Candida *spp*.* ^30^ and *S. mutans* ^3^, respectively. However, the solutions were employed along with coconut soap or as overnight immersions.

The main component of *R. communis* oil solution is sodium ricinoleate, which may inhibit biofilm formation^4^; though its mechanism of action is still unknown. The detergent action of this solution against microorganisms causes cell wall damage that results in the loss of cytoplasmic components and consequently cell death^4^. Endodontic studies evaluating 3.3% castor oil-based detergent showed antimicrobial activity of this product against anaerobes and streptococci present in teeth showing pulp necrosis^9^ and its effectiveness in cleaning root canals^15^. Other studies evaluating the antimicrobial activity of castor-oil based detergent - used for irrigating root canals - have verified its effectiveness against gram-positive microorganisms present in endodontic infections^12^.

Few studies have evaluated the efficacy of castor oil-based products as complete denture cleansers. Previous works have studied a 2% solution and although the product had not caused significant adverse effects on the properties of artificial teeth and acrylic resin^20^ ^,^ ^21^, it has been shown, by randomized clinical trials, moderate efficacy on denture biofilm removal^1^ ^,^ ^14^. Pinelli, et al.^19^ (2013) evaluated the effectiveness of a castor-oil mouthwash (Perioquil) in the treatment of institutionalized complete denture wearers with denture stomatitis and found a reduction of the disease’s clinical signs, however, there was no significant reduction in CFU/mL. An *in vitro* study showed that a 10% *R. communis* oil immersion solution, used for 20 min, provided moderate efficacy against tested species, as *S. mutans*, *Staphylococcus aureus*, *Escherichia coli*, *Pseudomonas aeruginosa*, *C. albicans* and *C. glabrata*, with the effective action on *Bacillus subtilis* and non-significant action on *Enterococcus faecalis* ^25^.

The antimicrobial activity of *R. communis* oil solution against *S. mutans* and *Candida *spp. was a relevant result because both these microorganisms cause denture stomatitis^24^. These results are in agreement with Malheiros-Segundo, et al.^14^ (2014) who found a reduction of *Candida* species (*C. albicans, C. glabrata, C. dubliniensis, C. krusei* e *C. tropicalis*) and *S. mutans* when a 2% solution was employed. As in previous study^12^, the solution was ineffective against gram-negative bacteria; such results can be explained by the structural characteristics of these microorganisms, which have a cell wall with another membrane, acting as a barrier to penetration of substances such as antiseptics and antibiotics^26^.

Studies have reported the antimicrobial activity of plants and seeds used as raw materials for manufacturing soaking solutions. These products are relatively safer synthetic alternatives and offer significant and more affordable therapeutic benefits. *R. communis* grows in several countries, which makes its trade and availability easier for complete denture wearers in the future. *R. communis* oil solution is colorless and does not have an unpleasant smell. These characteristics along with detergent action make its use in denture cleaning possible. Moreover, the immersion method for cleaning dentures is beneficial because it is easy and can be employed as an auxiliary method along with brushing.

Different species of fungi have been related to the development of denture stomatitis and these microorganisms have been found in immunocompromised patients and in healthy subjects^5^. *Candida* spp. isolated in this study was identified as *C. albicans*, *C. tropicalis*, *C. glabrata*, *C. parapsilosis* and *C. lusitaniae.C. albicans* was the most frequently isolated species, followed by *C. tropicalis* and *C. glabrata*. These species are commonly present in denture biofilms and are the main cause of atrophic chronic candidiasis^24^.

Tested solutions’ ability to prevent the formation of denture biofilms and their possible adverse effects on acrylic resins was not evaluated in the study. Therefore, future studies should include these analyses of concentrations employed in this study and in accordance with the established protocol.

## CONCLUSIONS

The obtained data indicated that 0.5% sodium hypochlorite solution was the most effective and might be used for short immersions along with brushing to control denture biofilm formation.

*C. albicans* was the most frequently isolated species from denture biofilms, followed by *C. tropicalis* and *C. glabrata*. Of the isolated strains, 18 (11.3%) were resistant to at least one of the tested antifungals, highlighting the resistance to azole compounds.
